# Graphene FET Sensors for Alzheimer’s Disease Protein Biomarker Clusterin Detection

**DOI:** 10.3389/fmolb.2021.651232

**Published:** 2021-03-26

**Authors:** Theodore Bungon, Carrie Haslam, Samar Damiati, Benjamin O’Driscoll, Toby Whitley, Paul Davey, Giuliano Siligardi, Jerome Charmet, Shakil A. Awan

**Affiliations:** ^1^Wolfson Nanomaterials and Devices Laboratory, School of Engineering, Computing and Mathematics, Faculty of Science and Engineering, University of Plymouth, Plymouth, United Kingdom; ^2^Division of Nanobiotechnology, Department of Protein Science, Science for Life Laboratory, School of Engineering Sciences in Chemistry, Biotechnology and Health, KTH Royal Institute of Technology, Stockholm, Sweden; ^3^Diamond Light Source, Rutherford Appleton Laboratory, Oxfordshire, United Kingdom; ^4^Institute of Digital Healthcare, WMG, University of Warwick, Coventry, United Kingdom

**Keywords:** graphene, field-effect transistors, biosensor, Clusterin protein, Alzheimer’s disease, SRCD absorbance spectroscopy, DNA and molecular diagnostics, cancer and cardiovascular disease detection

## Abstract

We report on the fabrication and characterisation of graphene field-effect transistor (GFET) biosensors for the detection of Clusterin, a prominent protein biomarker of Alzheimer’s disease (AD). The GFET sensors were fabricated on Si/SiO_2_ substrate using photolithographic patterning and metal lift-off techniques with evaporated chromium and sputtered gold contacts. Raman Spectroscopy was performed on the devices to determine the quality of the graphene. The GFETs were annealed to improve their performance before the channels were functionalized by immobilising the graphene surface with linker molecules and anti-Clusterin antibodies. Concentration of linker molecules was also independently verified by absorption spectroscopy using the highly collimated micro-beam light of Diamond B23 beamline. The detection was achieved through the binding reaction between the antibody and varying concentrations of Clusterin antigen from 1 to 100 pg/mL, as well as specificity tests using human chorionic gonadotropin (hCG), a glycoprotein risk biomarker of certain cancers. The GFETs were characterized using direct current (DC) 4-probe electrical resistance (4-PER) measurements, which demonstrated a limit of detection of the biosensors to be ∼ 300 fg/mL (4 fM). Comparison with back-gated Dirac voltage shifts with varying concentration of Clusterin show 4-PER measurements to be more accurate, at present, and point to a requirement for further optimisation of the fabrication processes for our next generation of GFET sensors. Thus, we have successfully fabricated a promising set of GFET biosensors for the detection of Clusterin protein biomarker. The developed GFET biosensors are entirely generic and also have the potential to be applied to a variety of other disease detection applications such as Parkinson’s, cancer, and cardiovascular.

## Introduction

Graphene, a single atomic plane of carbon, was considered to be thermodynamically unstable until 17 years ago. Novoselov et al. (2004) experimentally demonstrated that graphene can exist in the free state at room temperature, and that it is stable as a single layer of graphene making it a zero bandgap semiconductor. The monolayer of sp^2^ bonded carbon atoms is tightly packed into a two-dimensional (2D) sheet arranged in a honeycomb lattice. Graphene has the potential to advance many technological areas because of its outstanding material properties such as its high carrier mobility (Novoselov et al., 2004, 2005b; Bolotin et al., 2008; Morozov et al., 2008), current carrying capacity (Castro Neto et al., 2009), thermal conductivity (Balandin et al., 2008), optical properties (Blake et al., 2008), and mechanical stability (Booth et al., 2008). It is being researched for various applications such as high-speed electronics (Lin et al., 2010; Awan et al., 2016), optoelectronics (Bao and Loh, 2012), solar cells (Wang et al., 2008), energy storage (Wang et al., 2009), electromechanical resonators (Bunch et al., 2007), composites (Stankovich et al., 2006), and biosensors (Justino et al., 2017; Haslam et al., 2018; Vu and Chen, 2019).

Graphene is ideally suited to applications in biosensing due to its large surface-to-volume ratio, biocompatibility, chemical stability, ease of surface functionalisation, field effect-based ambipolar transport of electrons and holes, and excellent electrical conductivity (Geim and Novoselov, 2010), which are highly beneficial for good sensor performance such as increased sensitivity and a low limit of detection (LOD). Among the many graphene-based biosensor applications, graphene field-effect transistors (GFETs) are widely regarded as a promising platform for biosensing (Haslam et al., 2018; Bunch et al., 2020). The graphene channel in GFETs is typically exposed to the charged biological environment and is able to detect the presence of biomolecules electrically, based on resistance/conductance change caused by the binding of receptor molecules with a given antigen biomarker (enzymes, proteins, peptides, DNA, etc.). Dong et al. (2010), Okamoto et al. (2012), and Vu and Chen (2019). GFET is a powerful biosensing platform due to its relative simplicity in sensor preparation but also high signal-to-noise ratio, low-cost, portability, and relative ease of integration with a range of existing electronic systems. GFETs consume less energy, can be scaled down and can operate at higher frequencies making them a flexible platform for biosensing (Novoselov et al., 2004, 2005a; Awan et al., 2016). Such GFET biosensors are being researched extensively for early diagnosis of not only Alzheimer’s disease (AD) but also for a variety of other diseases such as Parkinson’s, cancer and cardiovascular.

Here, we report on the detection of Clusterin, a prominent protein biomarker of AD using both electrical and B23 absorption spectroscopy approaches. AD is a sub-type of dementia responsible for around 60–70% of cases in neurodegenerative diseases. There are approximately 54 million people currently living with dementia worldwide and this number is expected to rise to 130 million by 2050, and an estimated 9.9 million people will develop the disease every year (Alzheimer’s Society, 2020). AD is an incurable and long-term neurodegenerative disease that progressively worsens over time. It is believed to be caused by abnormal build-up of proteins in and around the brain. Tests have revealed deposits of protein around the extracellular and intracellular compartments of a post-mortem AD brain, the intracellular deposits were made up of filaments of hyperphosphorylated tau protein (Iqbal et al., 2005). Evidence has shown that neurofibrillary tangles (NFTs), consisting of hyperphosphorylated tau, are present within the neurons of AD patients; NFTs disrupt the normal communication between neurons (Selkoe, 1991). While the extracellular deposits also known as amyloid plagues are most commonly found in the neocortex (responsible for sensory perception, reasoning, conscious thoughts, generation of motor commands, and language in humans). The neocortex consists of 4-kDa polypeptide known as the β-amyloid (Aβ) (Glenner and Wong, 1984; Masters et al., 1985). Research has shown considerable evidence that neurodegeneration that occurs in AD patients is as a result of the accumulation and aggregation of Aβ. Aβ plaques are formed within the medial temporal lobe and also within the cerebral cortex of the brain tissue. This Aβ formation is attributed to the abnormal metabolism of β-amyloid precursor protein (APP) (Hardy and Selkoe, 2002). The Aβ plaques and NFTs develop over a long period of time (∼20–30 years) and leads to death of nerve cells and loss of brain tissues. Clusterin (is encoded by the single copy CLU gene located at the p21 – p12 locus on chromosome 8 in humans), also known as apolipoprotein-J, is a glycoprotein found in various tissues and bodily fluids (De Silva et al., 1990), and it functions as an extracellular chaperone (Satapathy, 2017). Increased levels of Clusterin have been found in the frontal cortex and hippocampus of post-mortem AD brain tissue (May et al., 1990; Lidström et al., 1998). It was demonstrated that CLU is strongly associated to soluble Aβ in the cerebral spinal fluid (CSF) (Ghiso et al., 1993), and it can either prevent the aggregation of Aβ or increase its solubility (Matsubara et al., 1996). It regulates the formation and toxicity of Aβ fibril, and also aids in transporting Aβ across the blood–brain barrier. Clusterin has a molecular weight of 75–80 kDa and is comprised of 449 amino acids (Tsuruta et al., 1990; James et al., 1991), two ∼40 kDa subunits of α and β connected by five disulphide bond motif (Kirszbaum et al., 1992). Clusterin plays an important role in the progression of AD and it has been identified as one of the key biomarkers of AD (Thambisetty et al., 2010). It was experimentally shown that Clusterin was elevated by ∼40% above non-demented controls in the brain of AD patients (May et al., 1990; Oda et al., 1994). Different Clusterin levels have been reported for AD patients; 67 AD cases were studied with mean age 85.3 ± 3.2 and Clusterin plasma levels of 158.5 ± 45.3 μg/mL (Schürmann et al., 2011), 60 AD cases were studied with mean age 83.4 ± 7.3 with Clusterin plasma levels of 129 ± 29 μg/mL (Schrijvers et al., 2011), also 17 AD cases with mean age 86.0 ± 6.36 and Clusterin plasma levels of 106.3 ± 23.7 μg/mL (Thambisetty et al., 2012). Currently, diagnosis of AD can take up to 2 years involving a range of different tests such as computer tomography (CT), magnetic resonance imaging (MRI), positron emission tomography (PET), neurological evaluation, cognitive, and neuropsychological tests (Mayo Clinic, 2020). Therefore, the need for a fast, low-cost, accurate, non-invasive, and portable means of diagnosing AD at an early stage is of high importance. Here we demonstrate the sensitivity, repeatability and specificity of the GFET biosensors for the detection of pure proteins. The next stage of the study will be to investigate the sensor response using real patient samples (such as serum, plasma, or blood), stability and storage of the sensors (Teixeira et al., 2014; Leva-Bueno et al., 2020; Zupančič et al., 2021). Thus, GFET biosensors offer a unique route towards the development of vitally needed diagnostic platform for AD.

## Materials and Methods

### Materials

Monolayer graphene was produced by chemical vapour deposition (CVD) method on a 300 nm Si/SiO_2_ substrate, supplied by Graphenea (San Sebastián, Spain) and LG Electronics Inc. (Gangseo-gu, Seoul, South Korea). The chemicals used for the GFET fabrication were photoresist, lift-off resist (LoR), Microposit developer and Microposit remover, purchased from A-Gas Electronic Materials (Warwickshire, United Kingdom).

Recombinant human Clusterin protein and anti-Clusterin antibody were purchased from Abcam (Cambridge, United Kingdom). Linker molecule 1-pyrenebutanoic acid succinimidyl ester (Pyr-NHS), bovine serum albumin (BSA) blocking solution and phosphate-buffered saline (PBS) were purchased from Sigma-Aldrich (Dorset, United Kingdom). All measurements were performed at room temperature, pH of 7.4 and using 10 μL samples deposited on the GFET sensors.

The quality of graphene was evaluated using an XPLORA Raman spectroscopy system (HORIBA, Northampton, United Kingdom). All measurements on the XPLORA system were performed at a wavelength of 532 nm, with ∼ 4 mW of incident power and a grating of 1200T. The XPLORA Raman system was interfaced with an OLYMPUS BX41 microscope (Shinjuku, Japan).

The electrical characterisation of the GFETs was performed under ambient conditions using a Keysight B1500A semiconductor device parameter analyser interfaced to a MPS150 probe station (Cascade Microtech, Thiendorf, Germany). The 4-probe current–voltage measurements (I_D_–V_D_ and I_D_–V_G_) were acquired as a function of gate voltage (V_G_) from −100 V to +100 V with I_D_–V_D_ curves from −50 to +50 mV with a 100 μA compliance. The I_D_–V_D_ output and I_D_–V_G_ transfer curves were measured at each functionalisation stage.

All absorption spectroscopy experiments were performed on Beamline B23 at Diamond Light Source (Oxfordshire, United Kingdom) over the 180–400 nm wavelength range, using a wavelength increment of 1 nm, with cell path length of 1 mm and at room temperature ∼ 22°C (Hussain et al., 2012).

### Fabrication of the Graphene FETs

**FIGURE 1:**
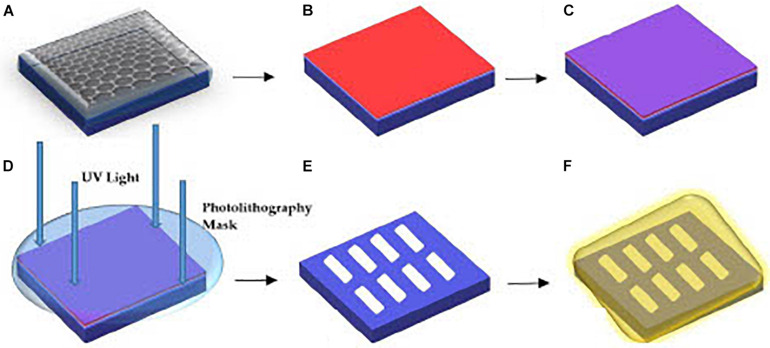
Overview of the processes for GFET fabrication **(A)** layer of graphene on Si/SiO_2_ substrate, **(B)** LoR deposition, **(C)** PR deposition, **(D)** sample below the photolithography mask aligner for UV exposure, **(E)** etching of graphene channels and chemical developing process, **(F)** final stage of metallic Cr and Au deposition.

The GFET sensors were fabricated on a p++ Si/SiO_2_ substrate through the processes of photolithographic patterning and metal lift-off techniques with evaporated chromium and sputtered gold contacts. There are two major stages in the fabrication process, the first stage involves forming the graphene channel whereas the second stage involves forming the source, drain, and voltage electrodes. A representation of the fabrication process is shown in Figure 1.

The formation of the graphene channels on the Si/SiO_2_ substrate involves dicing the samples into sizes of 1 cm × 1 cm chips before spin-coating the samples with LoR at 3000 revolutions-per-minute (rpm) for 30 s. The samples are then pre-baked in a fan oven at 175°C for 15 min. Pre-baking the samples solidify the LoR as it eases in lifting off the photoresist (PR) while protecting the graphene channels formed. The next step is spin-coating the samples with a layer of positive PR at 3000 rpm for 30 s and then post-baking on a hotplate at 100°C for 60 s. The post-bake step is to solidify the PR and remove any solvents on the samples. The samples are then positioned in a mask-aligner under a patterned mask for creating the graphene channels, and exposed to ultra-violet (UV) radiation for 25 s. The samples are then rinsed in a chemical developer mixed with di-ionized water (20 mL developer mixed with 30 mL di-ionized water), until the graphene channels are visible under a microscope. There is always residue of PR/PMMA (poly-methyl methacrylate) on the sample at this stage, which degrades the quality of the graphene, which can be reduced by post-baking the samples on a hotplate at 180°C for 8 min under deep ultraviolet (DUV) light of 254 nm. The DUV dissociates the bonds between PR/PMMA and graphene and reduces contact resistance of the sample.

Next, the samples are transferred into a sputtering machine for Ar plasma etching. The unprotected graphene samples (not protected by PR) are then etched by plasma formed from the ionization of Ar gas particles in a vacuum of 6 × 10^–7^ Torr at 50 W RF power for 2.5 min before the samples are treated with a chemical remover. Subsequently, the samples were placed in a chemical remover in an ultrasonic bath for ∼1 h at 60°C, the ultrasonic bath was turned off and the samples were left in the chemical remover for 15 h. The samples were then rinsed in di-ionised water and left to dry in a vacuum chamber for an hour.

The second fabrication stage involves forming metallic Cr and Au electrodes as the source, drain, and voltage electrodes. The electrode formation follows the same process as the graphene channel formation, but the samples are post-baked in an oven at 120°C for 15 min instead of exposing to DUV on a hotplate. Next, Cr is thermally evaporated using an Edwards Thermal Evaporator. The Cr target was heated to ∼2000°C for 8 s in a vacuum pressure of 10^–6^ Torr to form a 5 nm layer of Cr on the graphene samples. Thermal evaporation is a gentle way of depositing Cr on the samples without destroying the graphene channels already formed, and the Cr layer functions as an adhesive layer between graphene and the Au metallic contacts (Haslam et al., 2018). Using the Nordiko sputtering machine, 30 nm of Au was sputtered directly onto the Cr layer. The treatment with chemical remover was repeated and the samples were dried in a vacuum chamber for a further 1 hour.

**FIGURE 2:**
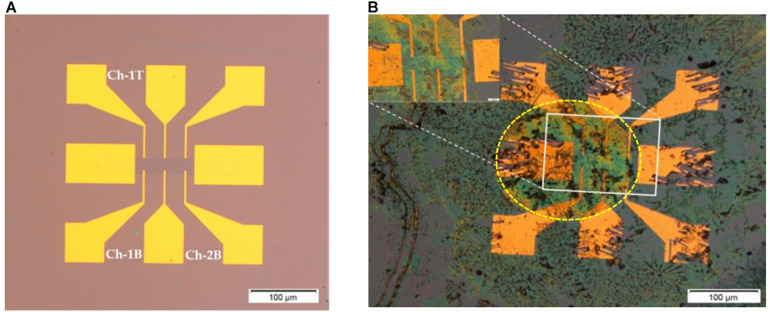
Graphene field effect transistor (GFET) device; **(A)** symmetric GFET device with graphene channel length of 400 μm (symmetry by 3 voltage leads above and below the graphene channel). Here Ch-1T refers to channel 1 top voltage leads, Ch-lB refers to channel 1 bottom voltage leads and Ch-2B refers to channel 2 bottom voltage leads; **(B)** GFET device with antibody bound Clusterin (at 1 pg/mL concentration), inset shows closer view of graphene channel and yellow dotted lines indicate the drop of solutions on the two series GFET channels (diameter of the drop region is ∼900 μm).

The fabricated 1 cm × 1 cm chips consists of 15 GFET devices, 5 asymmetric GFETs with the graphene channel length of 720 μm and 10 symmetric GFETs with graphene channel length of 400 μm with both being 80 μm (Haslam et al., 2018). Overall, the GFET sensors take approximately 3 days to fabricate and a further 1–2 days for biofunctionalisation and characterisation of the sensors. Figure 2 shows two symmetric GFET sensors in series in a device (deployed in this study) and when they are exposed to Clusterin at a concentration of 1 pg/mL.

### Functionalisation of GFETs

**FIGURE 3:**
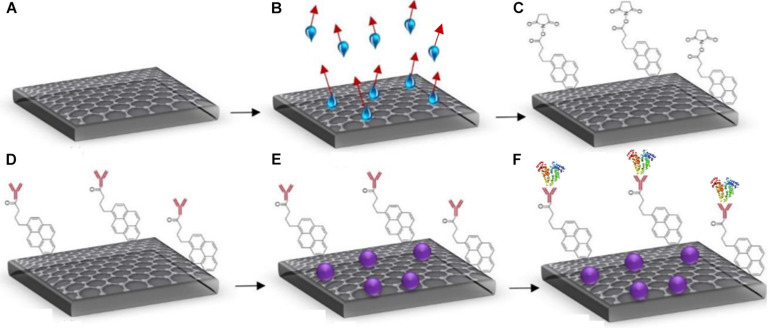
Steps for GFET functionalisation **(A)** bare graphene, **(B)** annealed graphene (showing evaporation of water molecules), **(C)** attachment of Pyr-NHS ester molecules with graphene, **(D)** anti-Clusterin antibody attachment to linkers (red), **(E)** BSA blocking (purple), and **(F)** binding of Clusterin (tri-colour molecules) with the anti-Clusterin antibody.

The GFETs were functionalised by immobilising the graphene surface with an anti-Clusterin antibody using a linker molecule, after which BSA was deposited and the final step involved conjugation of the Clusterin antigen in varying concentrations following the same protocol as Haslam et al. (2018). The steps involved in the biofunctionalisation processes are illustrated in Figure 3.

The Pyr-NHS ester linker molecules are a cross-linking agent that react with special functionalised groups such as amino groups on proteins. The linker molecule (1-pyrenebutanoic acid succinimidyl ester) used belongs to N-Hydroxysuccinimide (NHS) group known as Pyr-NHS ester. It has an aromatic pyrenyl group that strongly interacts with the surface of graphene via non-covalent π–π interactions and the succinimidyl ester group covalently reacts with the amino group (NH_2_) of the antibody (Huang et al., 2011). The linker molecule was applied at a concentration of 2 mM and was allowed to incubate at 4°C for 4 h, after which it was rinsed thrice with PBS and allowed to dry in ambient temperature. Once dry, Raman and electrical characterisation were performed followed by the binding of anti-Clusterin antibody. The antibody (Ab) was applied at a concentration of 20 μg/mL and was allowed to incubate following the same procedure as for the linker stage. BSA was then deposited on the GFET to block sites between the Ab regions preventing non-specific binding. BSA increases the tendency of the antibody to bind with the antigen of interest and it also improves the sensitivity of the sensor by decreasing background noise as the additional sites are blocked (Biocompare, 2012). BSA was deposited at a concentration of 0.5% and allowed to incubate following the same procedure as for the linker and Ab stages.

The final step involved depositing Clusterin antigen on the samples at varying concentrations from 1 to 100 pg/mL, followed by the samples being incubated for 1 h at 37°C before they were allowed to dry in ambient temperature and characterised using Raman spectroscopy and electrical 4-probe measurements (Awan et al., 2011).

## Results

### Characterization of GFETs

**FIGURE 4:**
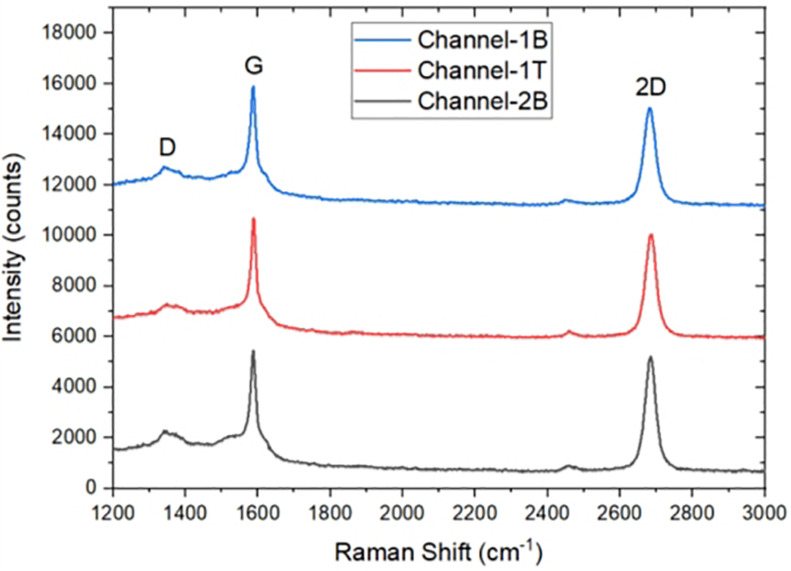
Raman Spectra of three monolayer graphene field-effect transistor (GFET) sensors after fabrication.

The GFETs were characterised using Raman Spectroscopy to analyse the quality of the graphene channels (Ferrari et al., 2006) and also with a semiconductor device parameter analyser to determine the electrical properties of the GFETs. Raman Spectroscopy is one of the most accurate, effective and non-destructive tool for the characterisation of graphene because of its sensitivity to important features and properties of graphene such as defect (Ferrari, 2007), doping (Casiraghi et al., 2007), strain (Huang et al., 2009), and temperature (Calizo et al., 2007). The Raman spectrum of graphene is made up of three main features with different physical origins; they are the 2D peak previously known as G′ peak, the G peak and D peak. In monolayer graphene, the 2D peak is observed at a Raman shift of ∼2700 cm^–1^, the G peak at a Raman shift of ∼1580 cm^–1^ and the D peak at a Raman shift ∼1350 cm^–1^ (Graf et al., 2007; Mafra et al., 2007). Figure 4 shows Raman spectra of three devices after fabrication, the intensity ratio I(2D)/I(G) position for Ch-1T = ∼ 1.10, Ch-1B = ∼ 1.04, and Ch-2B = ∼ 1.20 and full width half maximum (FWHM) of the 2D peak for Ch-1T = 59.8 cm^–1^, Ch-1B = 66.9 cm^–1^, and Ch-2B = 54.9 cm^–1^, which confirms the graphene channels are monolayer, and the D peak confirms the presence of defects, which is generally caused by the fabrication process.

**FIGURE 5:**
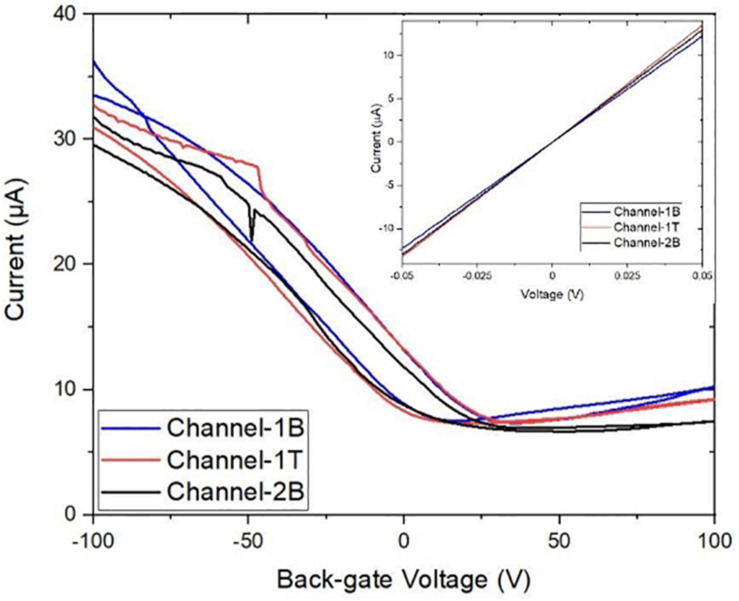
Characteristic transfer curve I_D_–V_G_ measurements of three graphene channels with corresponding output I_D_–V_D_ curves (inset). Back-gated measurements showing forward and reverse sweep (from –100 to 100 V) demonstrate the bare graphene channels are electrically almost identical.

The GFETs were electrically characterised using a Keysight B1500A semiconductor device parameter analyser interfaced to a Cascade probe station in ambient temperature and a four-probe I_D_–V_D_ and I_D_–V_G_ measurements were taken to study the electrical properties and performance of the sensors such as its sheet resistance, contact resistance, Dirac curve and mobility. I_D_–V_D_ measurements were taken on each sensor with a voltage sweep from −50 mV to +50 V and compliance of 100 μA. The sensors showed a linear response in current from changes in drain voltage, showing the channels are Ohmic as shown in the inset of Figure 5. Back-gated measurements were also performed to obtain the I_D_–V_G_ curve with a forward and reverse voltage sweep from −100 to +100 V, with a fixed drain voltage of 50 mV, Figure 5 (main panel). Data analysis was performed using the SCRAMBLE software which was developed in-house and graphs were plotted in Originlab. The I_D_–V_G_ curves show the Dirac curve of the graphene channels, revealing that the three channels are hole doped. From the curves, the carrier mobility of the three channels is found to be around 500–600 cm^2/^Vs (Zhong et al., 2015; Bøggild et al., 2017). Table 1 shows the Dirac point voltages (forward and reverse) and resistances for all three channels.

**TABLE 1:** The forward and reverse Dirac point voltages (DPV) and the resistance of the three channels. The Dirac point voltages reveal that the GFET is p-doped. Back-gate sweep rate was ∼ 20 V/s.

Channel	Forward sweep	Reverse sweep	Resistance
Channel-1B	17 V	35 V	4086 Ω
Channel-1T	35 V	22 V	3789 Ω
Channel-2B	42 V	51 V	3852 Ω

**FIGURE 6:**
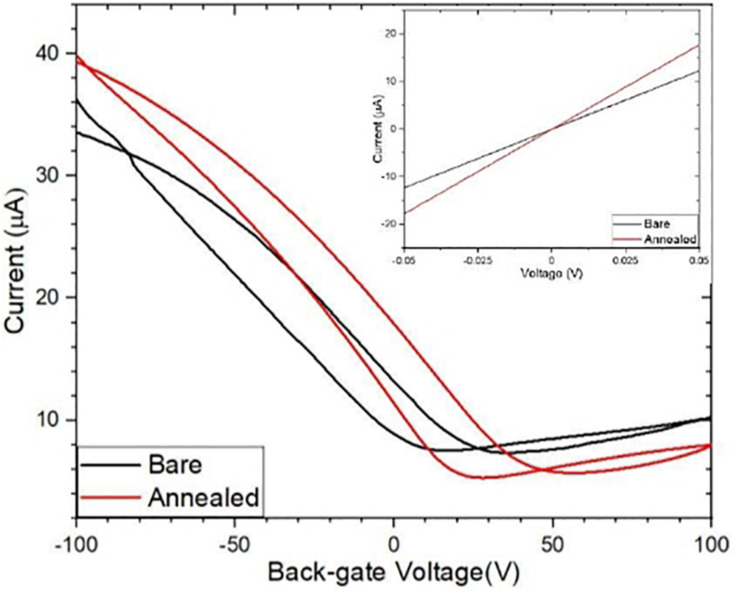
Comparison of the bare and annealed I_D_–V_G_ curves with their corresponding I_D_–V_D_ curves (inset).

During the transfer of CVD graphene onto a substrate, a polymer PMMA is used to support the graphene while the Cu substrate is etched. The PMMA often leaves a layer of residue on the graphene surface and the residue remains on the surface even after fabrication. The standard process of removing PMMA using acetone does not completely remove the residues because of strong Van der Waals interaction with graphene (Cheng et al., 2011). The presence of the residue and also water molecules from the graphene surface degrade the transport properties of graphene, causing a weak p-doping (Lin et al., 2012). We explored the effect of annealing to remove these contaminants from the surface of graphene, thermally annealing GFETs improve their performance increasing carrier mobility of the graphene channels (Cheng et al., 2011; Pirkle et al., 2011; Kumar et al., 2013). We annealed the GFET at 215°C for 30 min. Figure 4 shows results for Channel-1B at bare stage and after annealing. Figure 6 shows results for Channel-1B at bare stage and after annealing.

### Functionalisation of GFETs

**FIGURE 7:**
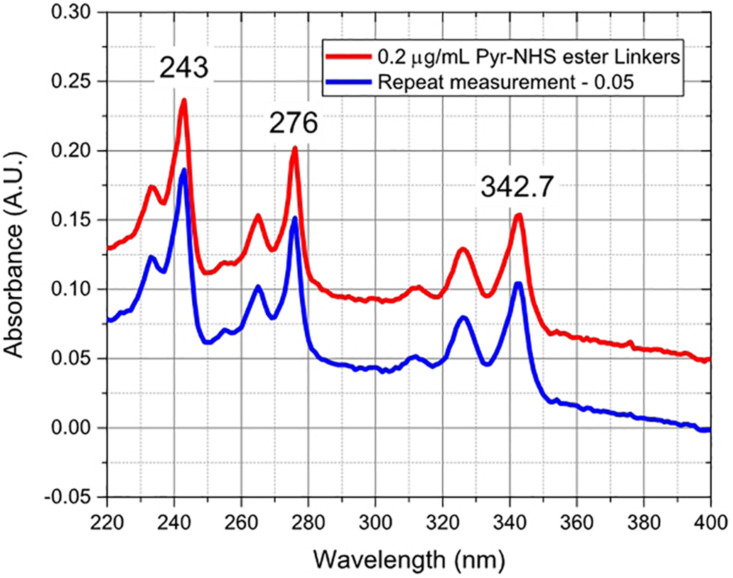
Absorbance spectra of Pyr-NHS ester linker molecules at 0.2 μg/mL concentration (red curve) and a repeat measurement (blue curve) off-set by 0.05 for clarity, showing the characteristic triple peaks due to the presence of pyrene moiety.

After annealing, the resistance of the device decreased by 31% from ∼4086 to ∼2822 Ω, and the carrier mobility increased by ∼ 43% from 460 to 660 cm^2^/Vs, improving the performance of the sensor. The annealed GFETs were then exposed to Pyr-NHS ester linker molecules using the drop-cast method, as shown in Figure 2B where the diameter of the solution drop is typically ∼ 1 mm. The linker molecules were also characterised using absorbance spectra, which could easily resolve a concentration level of 0.2 μg/mL, as shown in Figure 7. The data show clear triple peaks in absorbance due to the pyrene moiety that is central to the Pyr-NHS ester linker molecules, also observed by,Baek et al. (2011) after functionalising carbon nanotubes for electrical detection of DNA hybridisation. The data in Figure 7 also show a repeat measurement (off-set for clarity) of the absorbance spectra over the 220–400 nm wavelength range, demonstrating excellent repeatability of the measurements. The independent absorbance spectra essentially served to increase our confidence in the linker solutions we deployed for the GFET sensor experiments.

### Detection of Analytes Using GFETs

**FIGURE 8:**
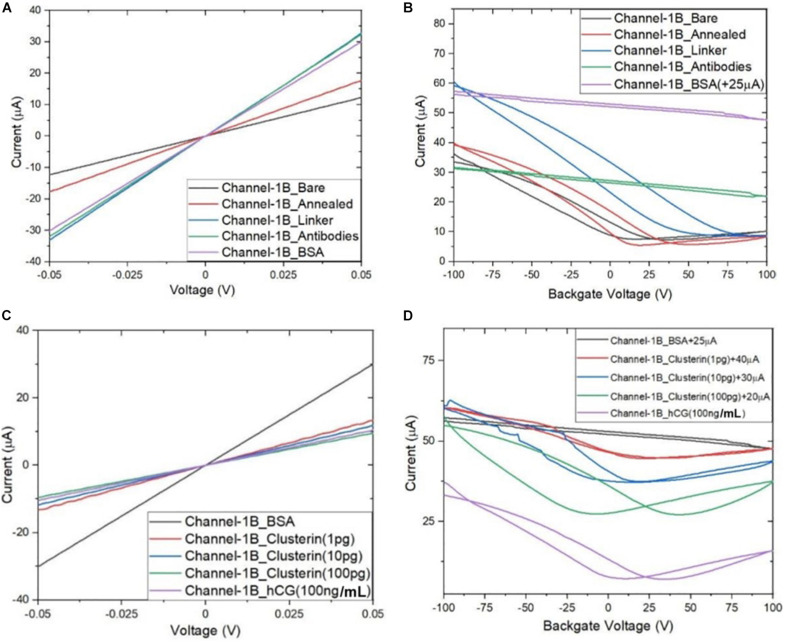
Characteristic curves for Channel-1B **(A)** I_D_–V_D_ output curves for bare to BSA, **(B)** I_D_–V_G_ transfer curves for bare to BSA, **(C)** I_D_–V_D_ curves for BSA to human chorionic gonadotropin (hCG) (100 ng), and **(D)** I_D_–V_G_ curves for BSA to hCG (100 ng) with off-set currents shown for clarity. Antigen concentrations are in units of g/mL.

**FIGURE 9:**
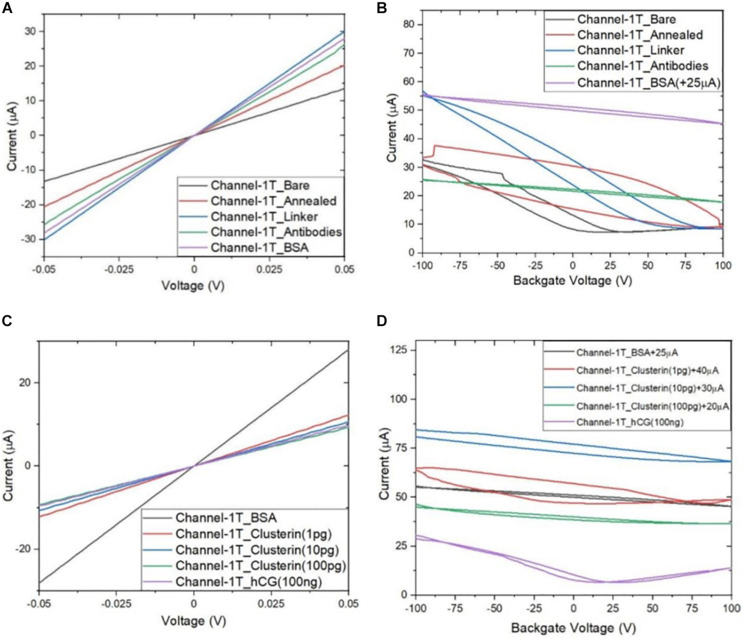
Characteristic curves for Channel-1T **(A)** I_D_–V_D_ output curves for bare to BSA, **(B)** I_D_–V_G_ transfer curves for bare to BSA, **(C)** I_D_–V_D_ curves for BSA to hCG (100 ng/mL), and **(D)** I_D_–V_G_ curves for BSA to hCG (100 ng/mL), with off-set currents shown for clarity. Antigen concentrations are in units of g/mL.

**FIGURE 10:**
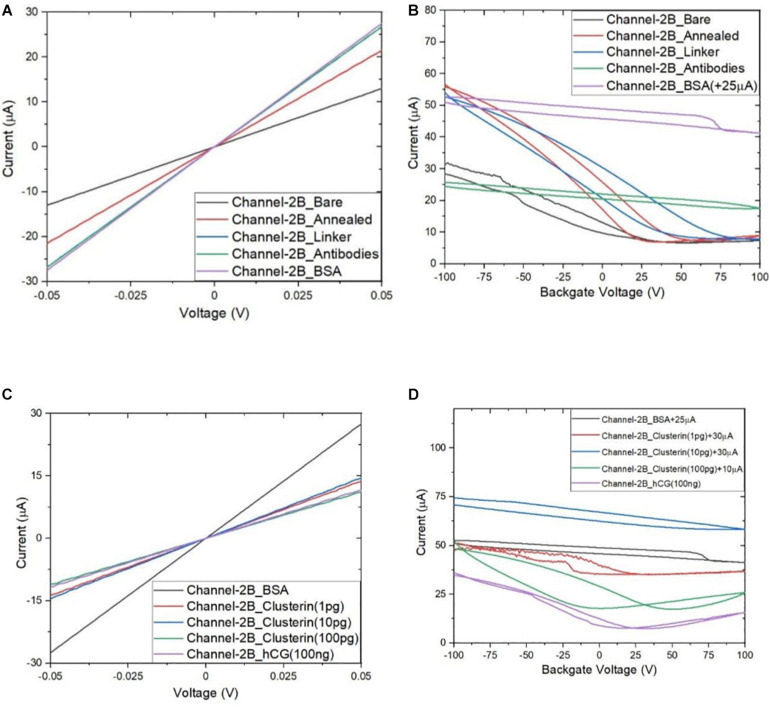
Characteristic curves for Channel-2B **(A)** I_D_–V_D_ output curve for bare to BSA, **(B)** I_D_–V_G_ transfer curve for bare to BSA, **(C)** I_D_–V_D_ curve for BSA to hCG (100 ng/mL), and **(D)** I_D_–V_G_ curve for BSA to hCG (100 ng/mL), with off-set currents shown for clarity. Antigen concentrations are in units of g/mL.

Following the functionalisation of the GFETs, Figures 8–10 show data from Channel-1B, Channel-1T, and Channel-2B for I_D–_V_D_ and I_D–_V_G_ characteristic curves. Figures 8A,B shows data for bare to BSA functionalisation stages, with I_D_–V_D_ showing linear Ohmic response and I_D–_V_G_ showing shifts in the Dirac point from bare to linker stages. However, it is interesting to note the Dirac points are at approximately 100 V limit of our measurement system for antibody to BSA stages. Although the I_D–_V_G_ characteristic curves provide quantitative indication of the influence of charged molecules on the surface of graphene, albeit with a relatively high standard deviation, at present a more robust quantitative response of the GFET sensors can be determined from the 4-probe electrical resistance (4-PER) measurements of the I_D–_V_D_ curves. In contrast, Figure 8C shows the standard ohmic response for functionalisation stages from BSA to Clusterin (at 1 to 100 pg/mL concentration levels) and 100 ng/mL of human chorionic gonadotropin (hCG), whereas Figure 8D shows the Dirac point is approximately constant around 25 V. This is in agreement with our previous observations (Haslam et al., 2018) that the Dirac point shift is typically in the few-volt region for similar concentrations of hCG and is significantly influenced by hysteresis and charge traps in the SiO_2_ substrate. Figures 9, 10 show almost identical response of the GFETs from bare to Clusterin antigen and hCG antigen detection. Table 2 shows the corresponding data from the GFET sensors from bare to Clusterin and hCG antigen stages based on the I_D–_V_D_ results.

**TABLE 2:** Measured resistance and resistance change (%) for bare to BSA stages of functionalisation for the three channels and the corresponding mean and standard deviation values.

Channel	Bare (Ω)	Annealed (Ω)	ΔR (%)	Linker (Ω)	ΔR (%)	Antibodies (Ω)	ΔR (%)	BSA (Ω)	ΔR (%)
Channel-1B	4086	2822	−31	1509	−47	1566	+3.8	1660	+6
Channel-1T	3789	2422	−36	1660	−31	1936	+16	1775	−8
Channel-2B	3852	2323	−35	1868	−20	1862	−0.3	1862	−2.4
Mean ± SD	3909 ± 128	2522 ± 216	−34 ± 2	1679 ± 147	−33 ± 11	1788 ± 160	6.5 ± 7	1766 ± 83	−1 ± 6

*The percentage changes are calculated for the two nearest stages (i.e., bare to annealed, annealed to linker, etc.).*

## Discussion

**FIGURE 11:**
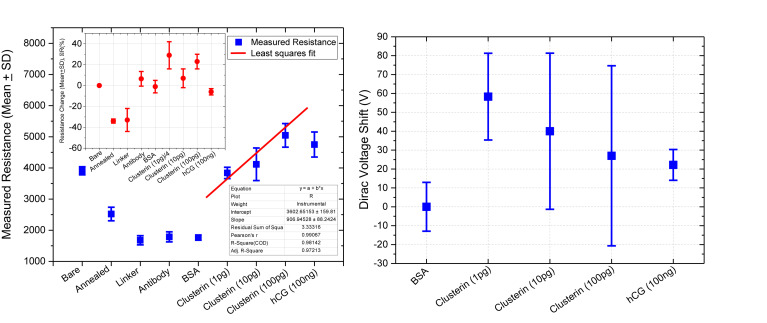
Measured resistance (in ohms, Mean ± SD) for each functionalization stage with a log-linear least squares fit (solid line, effectively a partial Hill–Langmuir equation fit) to the measured data from 1 to 100 pg/mL of Clusterin concentration and the fit parameters showing adjusted *r*^2^ ~ 0.98 **(left)**. The inset shows corresponding percentage resistance change relative to the previous stage (with bare stage being 0% by definition). Note, the data in the inset for resistance change at 1 pg/mL Clusterin is reduced by a factor of 4 for clarity and analyte concentrations are in units of g/mL. Corresponding back-gated measurements of Dirac voltage shifts normalized with respect to the BSA stage, demonstrating a first-time observation of a characteristic response curve for the GFET biosensors **(right)**.

Figure 11 shows plot of resistance change (Mean ± SD) for each channel at all stages of the functionalisation process. The data show that there is an approximate 30% reduction in resistance, from bare stage when the channels are annealed (inset in Figure 11). A further 30% reduction in resistance is observed when the linker molecules are bound to the graphene surface. In contrast, there is almost a negligible change in resistance when the antibodies and BSA interface with the linker functionalized GFETs. However, when 1 pg/mL of Clusterin is conjugated with the antibodies on the GFETs, we observe an ∼118% increase in resistance. We estimate a LoD ∼300 fg/mL (4 fM) for Clusterin detection, similar to the LoD demonstrated for hCG detection by Haslam et al. (2018) using our DC 4-PER measurement technique. The LoD was estimated using LoD ∼ 3.3 (σ/S), where σ is the standard deviation at low concentration (in our case at the BSA stage) and S is the sensitivity coefficient or slope of the calibration curve (over the log-linear dynamic range) shown in Figure 11. The log-linear least squares solid line fit to the data shown in Figure 11 is effectively a partial fit of the Hill–Langmuir equation (Hill, 1910; Langmuir, 1918). Figure 11 also shows a corresponding comparison with back-gated measurements of Dirac voltage shifts normalized with respect to the BSA stage. At 1 pg/mL of Clusterin concentration the Dirac voltage shift of approximately 60 ± 22.9 V, from the BSA stage, is clearly discernable (*p* < 0.001). At subsequent concentrations of Clusterin, 10 and 100 pg/mL, a clear reduction in the Dirac voltage shifts are observed. However, the standard deviations of the measurements are relatively large, as also observed by Tsang et al. (2019), and point to a future requirement for further optimisation of our next generation of GFETs and their fabrication processes. The initial 60 ± 22.9 V increase in Dirac voltage shift at 1 pg/mL Clusterin concentration and the subsequent gradual reduction in the Dirac voltage shift for 10 and 100 pg/mL indicate a reduction in the number of available binding sites (anti-Clusterin antibodies) on the GFET sensors. Thus, to the best of our knowledge, this is the first time such a characteristic curve (Figure 11, graph on the right) for Dirac voltage shift has been demonstrated for Clusterin detection using CVD single layer GFETs that may also broadly be expected for the detection of other molecular species. Table 3 shows a variety of biosensing platforms and detection techniques in comparison with our results reported in Table 4 using 4-PER and Dirac voltage shift techniques.

**TABLE 3:** Measured resistance and resistance change (%) for Clusterin (1 pg/mL) to hCG (100 ng/mL) stages of analyte detection for the three channels and the corresponding mean and standard deviation values.

Channel	Clusterin (1 pg) (Ω)	ΔR (%)	Clusterin (10 pg) (Ω)	ΔR (%)	Clusterin (100 pg) (Ω)	ΔR (%)	hCG (100 ng) (Ω)	ΔR (%)
Channel-1B	3778	+127	4225	+11	5212	+23	4754	−9
Channel-1T	4084	+130	4697	+15	5406	+15	5244	−2
Channel-2B	3657	+101	3429	−6	4524	+32	4257	−6
Mean ± SD	3840 ± 180	117 ± 13	4117 ± 523	7 ± 9	5047 ± 378	23 ± 7	4752 ± 403	−6 ± 3

*The Clusterin and hCG concentrations are in units of g/mL.*

**TABLE 4:** Other approaches deployed for biosensors in comparison with our GFET based four-probe electrical resistance (4-PER) detection technique.

Electrode materials	Receptor system	Detection technique	LoD (pg/mL)	References
SPCE-NPAu	SPCE-NPAu/Streptavidin/Biotin-Aβ-42/anti-Aβ/anti-IgG-AP	CV	100	Rama et al., 2014
Gold nanoparticles	GNP/MUA/NHS-EDC/Aβ(1–42) monoclonal antibody IgG/BSA/Aβ(1–42) peptide solution	EIS	1	Wu et al., 2014
SPCE/carbon	SPCE/PANHS/anti-hCG Ab/BSA/hCG	CV/electrochemical	1	Damiati et al., 2019
Au nanoparticles	Au/PSA antibody/BSA/PSA/tPSA	SPR	30	Uludag and Tothill, 2012
SPCE	SPCE/Pyr-NHS/anti-CLU F(ab′)_2_/BSA/CLU	CV/SWV	1	Islam et al., 2018
SPR chip-gold	Gold film/EDC-NHS/anti-cTnT antibody/BSA/cTnT	SPR	500	Pawula et al., 2016
Gold nanoparticles	Gold electrode/AuNP/MPA self-assembly/EDC-NHS/BSA/HER2	EIS	500	Chun et al., 2013
Graphite electrodes	Electrode/EDC-NHS/anti-CA125, anti-CA153, anti-CEA/BSA/CA125, CA153, CEA/M-Pt-CA125Ab_2_, M-Pt-CA153 Ab_2_, M-Pt-CEA Ab_2_	DPV	7	Cui et al., 2014
PDMS/AuNP	PDMS/AuNP/anti-human IgG(cTnI)/BSA/human IgG(cTnI)	Colorimetric	10	Wu et al., 2010
GFET	Graphene/Pyr-NHS/anti-CLU/BSA/CLU	4-PER	0.3	This work

We also tested the three GFET sensors for their specificity by introducing a three-orders-of-magnitude higher concentration (compared to 100 pg/mL of Clusterin) hCG antigen at a concentration of 100 ng/mL; data are presented in Tables 2, 3 and a summary of the results are shown in Figure 11. The three sensors resulted in only an average of −6 ± 3% change in resistance, demonstrating the excellent specificity of our GFET sensors and the functionalisation protocols. These highly promising results demonstrate the potential of our graphene sensors as low-cost, repeatable, sensitive, and specific detection platforms suitable for detecting a variety of other disease diagnosis. Our future work involves the development of a novel multiplexing platform exploiting the fact that these GFET sensors are generic transducers of biological events.

## Conclusion

We have reported the fabrication, functionalization, and characterization of graphene FET sensors using Raman spectroscopy, four-probe electrical measurements and absorbance spectra using the highly collimated microbeam of Diamond B23 beamline for the detection of a prominent AD protein biomarker, Clusterin. The fabrication and functionalisation protocols have enabled detection of Clusterin from 1 to 100 pg/mL, with a limit-of-detection of ∼300 fg/mL (4 fM) using 4-PER measurement technique. In contrast, a characteristic curve for the Dirac voltage shift with Clusterin concentration has also been demonstrated using back-gated I_D–_V_G_ measurements, although the standard deviation of these results were relatively higher than the results from 4-PER measurements. The GFET sensors were also found to have a repeatable performance over an extensive range of functionalisation stages from bare to 100 pg/mL of Clusterin using 4-PER measurements. In addition, the sensors were found to be highly specific, showing only a −6 ± 3% resistance change compared to 100 pg/mL of Clusterin when a three-orders-of-magnitude higher concentration of hCG was applied (100 ng/mL) to the GFET sensors. Future work includes deploying the sensors to detect a panel of biomarkers for the early detection of AD (such as ApoE, Aβ, etc.) using a novel multiplexing platform we are currently developing, which will be reported in a future study. In addition, as the GFET sensors are generic transducers, we anticipate their future applications in a variety of other disease biomarker detection also, such as cancer and cardiovascular.

## Data Availability Statement

The original contributions presented in the study are included in the article/supplementary material, further inquiries can be directed to the corresponding author.

## Author Contributions

SAA: conceptualization, formal analysis, data curation, project administration, and funding acquisition. SAA, TB, CH, SD, and BO’D: methodology. BO’D and TB: software. SAA, TB, GS, and JC: validation. SAA and TB: writing—original draft preparation. TB, CH, SD, BO’D, TW, PD, GS, JC, and SAA: writing—review and editing. SAA, TW, and PD: supervision. All authors have read and agreed to the published version of the manuscript.

## Conflict of Interest

The authors declare that the research was conducted in the absence of any commercial or financial relationships that could be construed as a potential conflict of interest.
